# Multi-Locus Estimates of Population Structure and Migration in a Fence Lizard Hybrid Zone

**DOI:** 10.1371/journal.pone.0025827

**Published:** 2011-09-29

**Authors:** Adam D. Leaché

**Affiliations:** Department of Biology and Burke Museum, University of Washington, Seattle, Washington, United States of America; Montreal Botanical Garden, Canada

## Abstract

A hybrid zone between two species of lizards in the genus *Sceloporus* (*S. cowlesi* and *S. tristichus*) on the Mogollon Rim in Arizona provides a unique opportunity to study the processes of lineage divergence and merging. This hybrid zone involves complex interactions between 2 morphologically and ecologically divergent subspecies, 3 chromosomal groups, and 4 mitochondrial DNA (mtDNA) clades. The spatial patterns of divergence between morphology, chromosomes and mtDNA are discordant, and determining which of these character types (if any) reflects the underlying population-level lineages that are of interest has remained impeded by character conflict. The focus of this study is to estimate the number of populations interacting in the hybrid zone using multi-locus nuclear data, and to then estimate the migration rates and divergence time between the inferred populations. Multi-locus estimates of population structure and gene flow were obtained from 12 anonymous nuclear loci sequenced for 93 specimens of *Sceloporus*. Population structure estimates support two populations, and this result is robust to changes to the prior probability distribution used in the Bayesian analysis and the use of spatially-explicit or non-spatial models. A coalescent analysis of population divergence suggests that gene flow is high between the two populations, and that the timing of divergence is restricted to the Pleistocene. The hybrid zone is more accurately described as involving two populations belonging to *S. tristichus,* and the presence of *S. cowlesi* mtDNA haplotypes in the hybrid zone is an anomaly resulting from mitochondrial introgression.

## Introduction

Studies of lineage divergence and the subsequent processes that occur when lineages reconnect and merge are greatly facilitated by investigations of naturally occurring hybrid zones. Hybrid zones are areas where two incompletely separated lineages overlap spatially and temporally and interbreed to form viable and at least partially fertile offspring [1]. North American phrynosomatid lizards in the genus *Sceloporus* have a history of being focal species in studies of hybridization and phylogeography [2], [3]. A hybrid zone involving *S. tristichus* and *S. cowlesi* located along grassland and juniper habitat ecotones on the Mogollon Rim in Arizona involves complex interactions between incompletely separated lineages with varying levels of divergence in morphology, chromosomes and mitochondrial DNA (mtDNA) [4]. Multiple processes are operating simultaneously in this hybrid zone, including the hybridization between closely-related yet strongly differentiated clades belonging to *S. tristichus*, and secondary contact between deeply divergent yet morphologically cryptic mitochondrial DNA (mtDNA) lineages belonging to *S. tristichus* and *S. cowlesi*.

**Figure 1:**
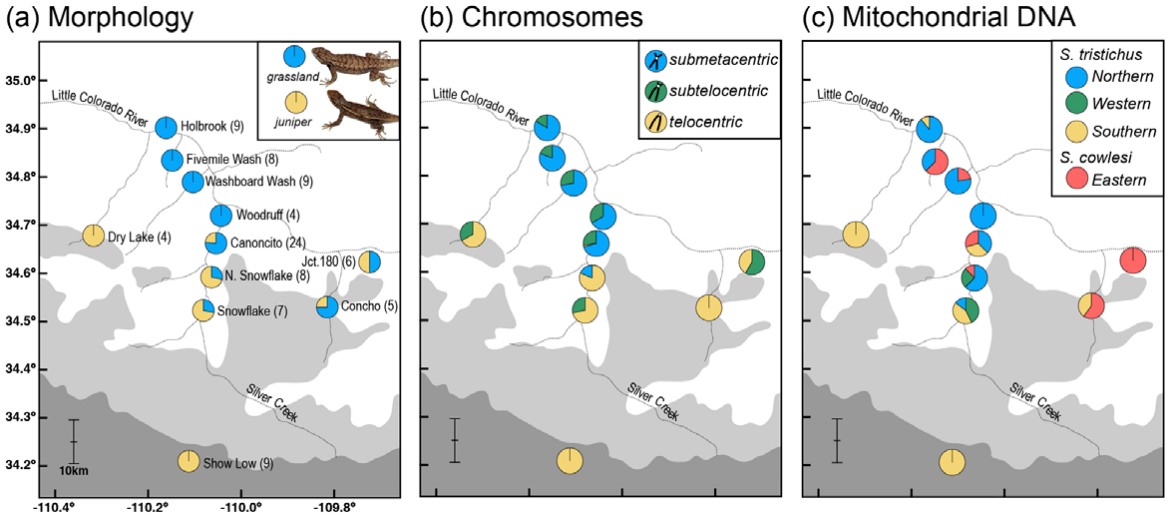
Character variation in the hybrid zone. Geographic variation in (a) morphology, (b) chromosomes, and (c) mitochondrial DNA haplotypes in the *Sceloporus* hybrid zone in Navajo County, Arizona, and the number of individuals sampled at each locality (a). The distributions of Petran Montane Conifer Forest, Great Basin Conifer Woodland, and Great Basin Grassland habitats are shown in dark grey, light grey, and white, respectively.

The populations at opposite ends of the hybrid zone are distinguishable using a combination of characters from morphometrics, scalation, and coloration [4]. These differences have historically been partitioned using a subspecies taxonomy to reflect the adaptive nature of populations [5]. The populations that inhabit the desert grassland regions at the northern end of the contact zone ( =  *elongatus*) differ markedly in size, coloration, behavior, and scalation from those that occur in the Pinyon-Juniper habitat to the south of the contact zone ( =  *tristichus*). This suggests that the hybrid zone follows a typical 2-population model, but two measures of genetic variation suggest that the hybrid zone may be more complex (Fig. 1).

A pericentric inversion polymorphism on chromosome seven provides one source of genetic variation for investigating the hybrid zone [6]. This polymorphism is defined by the location of the centromere along the chromosome, and three character states are found in the hybrid zone [4]. Specimens are usually homozygous for a single state, but hybrid specimens have heteromorphic pairs of chromosome seven (Fig. 1). The second source of genetic variation stems from mtDNA sequence data, and the hybrid zone contains haplotypes belonging to four distinct mtDNA clades [4]. These clades are partitioned into two putative species, *Sceloporus cowlesi* and *S. tristichus,* that differ by up to 10% uncorrected sequence divergence (Fig. 1) [7]. *Sceloporus cowlesi* is widespread throughout New Mexico and enters the contact zone from the east, presumably along the Little Colorado River [4]. The three remaining clades differ by up to 4.5% uncorrected sequence divergence and belong to *S. tristichus*, which is widespread in Arizona, northern New Mexico, Utah, and Colorado, and these clades enter the contact zone from the north, south, and west (Fig. 1) [4].

The spatial patterns of divergence in these characters are discordant across the hybrid zone, and the greatest morphological and chromosomal disparity is not between the most genetically divergent clades as might be expected [4]. The greatest mtDNA divergence is between *S. cowlesi* and *S. tristichus*, but most of the morphological and karyotypic divergence is found within *S. tristichus* mtDNA clades. Species trees for *S. cowlesi* and *S. tristichus* based on multi-locus nuclear data indicate that they are closely related [8] and not highly divergent and non-sister lineages as suggested by the mtDNA [7]. At the center of the hybrid zone, mtDNA haplotypes belonging to *S. cowlesi* are in cytonuclear disequilibrium with certain chromosomal rearrangements, indicating that some allelic combinations may experience epistatic effects on fitness or that nonrandom mating is occurring.

A fundamental question to address in this hybrid zone is whether multi-locus nuclear data corroborate the two distinctive morphological forms, the three chromosomal polymorphisms, or the four mtDNA clades. Mitochondrial introgression is believed to occur in the hybrid zone [4], [8], and therefore is a likely culprit for some of the observed discordance. Since no obvious extrinsic barriers to gene flow exist between populations across the hybrid zone, it is assumed that intrinsic barriers are responsible for impeding migration and subsequent gene flow between populations. A quantification of gene flow in the hybrid zone is also lacking. Here, coalescent analyses of the multi-locus data are used to estimate migration rates and divergence times between the inferred populations.

## Results and Discussion

### Multi-locus nuclear data

Population structure and gene flow were estimated from 12 anonymous nuclear loci collected for 93 specimens from throughout the hybrid zone (Supporting Information Table S1). The 12 nuclear loci contain 2 − 46 variable sites and 2 − 26 alleles, with longer loci generally containing more variation (Table 1). The population sample from the hybrid zone is weakly differentiated at the 12 nuclear loci (as indicated by F_st_ values; Table 1), and these low levels of differentiation are typical for slowly evolving nuclear loci and recently diverged lineages. The non-significant vales for Tajima's *D* indicate that none of the loci deviate from expectations of neutral evolution.

**Table 1:** Genetic variation of the 12 nuclear loci used in the study.

Locus	*N*	Length	Variable Sites	Alleles	Excluded	F_st_	Tajima's D
*Sun10*	90	285	17	20	7	0.01696	−0.97914
*Sun22*	93	385	13	13	5	0.12686	−1.06841
*Sun25*	93	140	3	3	0	0.62576	0.93026
*Sun27*	91	153	5	6	1	0.11931	−0.60739
*Sun28*	90	518	27	17	61	0.05299	−0.51682
*Sun31*	89	536	19	17	5	0.35638	−0.37215
*Sun32*	92	272	8	12	5	0.09673	1.33924
*Sun37*	93	620	46	26	13	0.04772	−1.53264
*Sun53*	83	399	25	20	8	0.06184	−1.17355
*Sun54*	81	40	5	2	0	0.00423	1.58029
*Sun59*	93	65	5	5	1	0.08360	−0.27152
*Sun60*	85	128	11	10	0	0.09709	−1.10006

*N* refers to the number of specimens sequenced.

Excluded haplotypes have PHASE reconstruction probability values <0.95.

F_st_ values are calculated using comparisons between northern and southern populations.

Tajima's D values are not significant for any locus.

### Population structure

**Figure 2:**
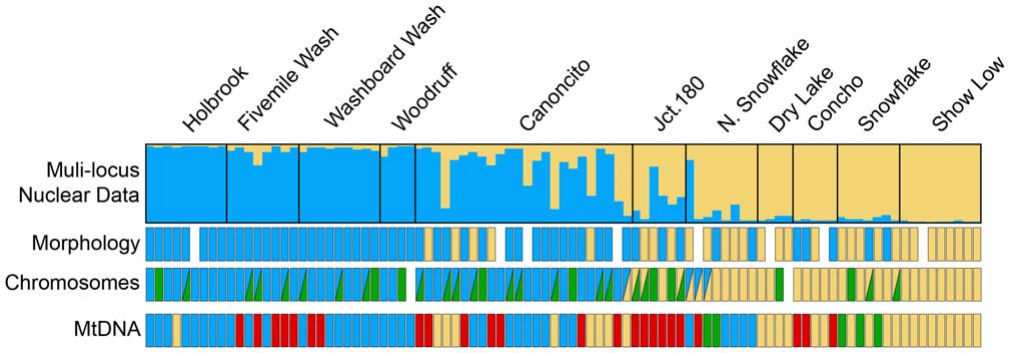
Population structure contrasted across character types. Estimated population structure across the hybrid zone based on the Structure analysis (*K* = 2) of the 12 nuclear loci. Each bi-colored vertical bar represents the estimated membership fraction of an individual into two population clusters. Assignments based on morphology, chromosomes, and mtDNA haplotypes are shown for comparison. Missing bars indicate missing data.

Population structure was estimated from the multi-locus nuclear data using Structure v2.2 [9] and Structurama v2.0 [10]. The number of populations in the hybrid zone estimated from the multi-locus nuclear data suggest that an increase in population clusters beyond *K* = 2 does not provide a good fit for the data (Table 2). The posterior probability for *K* = 2 is highest, and this estimate is robust to changes in the prior mean used in the Bayesian analysis (Table 2). The assignment of individuals into *K* = 2 population clusters produces a clear picture of the population structure across the hybrid zone (Fig. 2). The multi-locus genotypes for individuals in the four localities at the northern end of the hybrid zone (Holbrook, Fivemile Wash, Washboard Wash, and Woodruff) are grouped into one “northern” population cluster (Fig. 2). Individuals from southern localities (Show Low, Snowflake, and N. Snowflake), as well as those to the east and west of the hybrid zone (Concho and Dry Lake), are each composed of individuals with high fractions of “southern” genotypes (Fig. 2). Individuals are admixed at the localities at the center of the hybrid zone (Canoncito and N. Snowflake), and to the east (Fig. 2).

**Table 2:** Population structure in the *Sceloporus* hybrid zone based on 12 nuclear loci.

	Posterior Probability Distributions				Marginal Likelihoods
*K*	*E*(*K*) = 2	*E*(*K*) = 4	*E*(*K*) = 6	Spatially- explicit	Pr(X | *K*)
1	0.17	0.05	0.02	–	−2804.7
2	**0.77**	**0.73**	**0.60**	**0.68**	**−2674.6**
3	0.06	0.21	0.33	0.22	−2681.3
4	–	0.01	0.05	0.06	−2690.7

**Figure 3:**
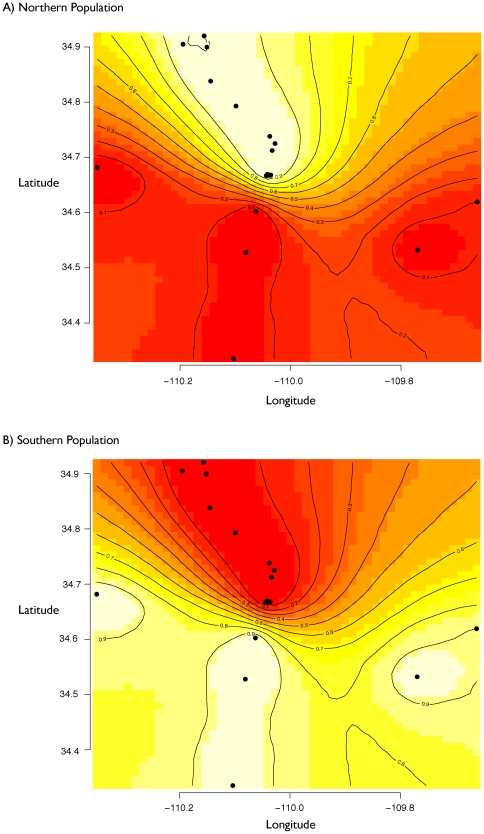
Spatially-explicit estimate of population clusters. Geneland maps of the *Sceloporus* hybrid zone with individual pixels assigned to population clusters (*K* = 2: A, northern population; B, southern population). The highest posterior probabilities are in white and the lowest are in red, and the contour lines depict the spatial change in population assignment probability.

In order to detect population subdivisions that take into account the spatial position of sampled genotypes, I used the Bayesian MCMC program Geneland [11], [12]. The MCMC analysis of population structure using Geneland places ∼70% of the posterior probability density on *K* = 2 population clusters, and the remaining posterior density is distributed across *K* vales from 3–5 in a descending fashion (Table 2). Thus, strong support is provided for a two population model, and the spatial depictions of the population memberships in the hybrid zone (at *K* = 2) are presented in Figures 3A and 3B. The 90% posterior probability contour encompassing the first population includes those same localities considered “northern” by the Structure analysis (Holbrook, Fivemile Wash, Washboard Wash, and Woodruff) as well as Canoncito, which is composed of a large number of admixed individuals (Fig. 3A). The remaining localities are assigned to a “southern” population, although a single locality (Jct. 180) is located in a region of slightly lower posterior probability (Fig. 3B). The center of the hybrid zone along Silver Creek is positioned just south of Canoncito, which is consistent with the maximum likelihood estimates of cline centers based on morphology and chromosomes [4].

Estimating population structure in the *Sceloporus* hybrid zone using multi-locus data provides a new framework for interpreting character discordance in this system. Although previous interpretations of the population structure of this hybrid zone differed depending upon which characters are examined, the population structure analyses of the 12 independent nuclear markers used in this study suggest that this hybrid zone is composed of two populations (Fig. 2; Table 2). These populations are distributed along a north-south gradient (Fig. 3). Interpreting the morphological, chromosomal, and mtDNA data collectively from the perspective of the nuclear population structure indicates that this hybrid zone involves two morphologically distinctive populations belonging to *Sceloporus tristichus*. Chromosomally, the submetacentric chromosome seven polymorphism dominates the northern population, whereas the telocentric chromosome polymorphism dominates in the south. The subtelocentric chromosome polymorphism is found throughout the interface of these populations (Fig. 2). The mtDNA genealogy supports two clades within *S. tristichus* that reflect the north-south population structure identified with the multi-locus nuclear data; however, the multi-locus nuclear data do not support the western *S. tristichus* mtDNA clade or the eastern *S. cowlesi* mtDNA clade as distinct populations. Previous studies have suggested that mtDNA introgression is occurring in the hybrid zone [4], [8], and the new multi-locus nuclear data analyzed here support this conclusion.

### Population divergence genetics

**Figure 4:**
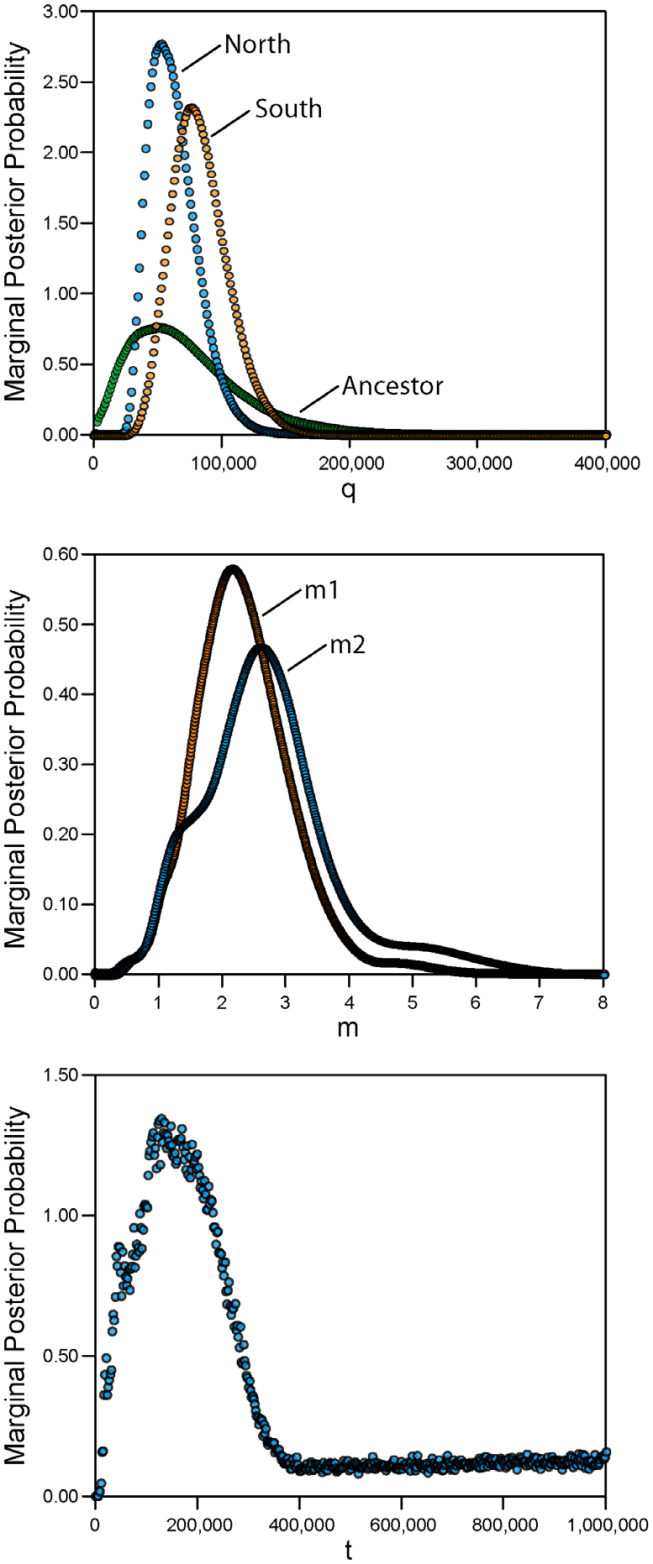
Population divergence genetic parameters. Marginal posterior probability distributions for the isolation-with-migration demographic parameters.

The migration rates, population sizes, and divergence time between the inferred populations were estimated using the isolation-with-migration program IMa2 [13]. Only those samples with assignments probabilities ≥ 0.95 were included in the analysis (Table 3). First, a recent divergence time (t) is supported by the data. The marginal posterior probability distribution for t shows a clear peak (high point) near 158,000 years (Fig. 4). The mean of the distribution is substantially older (696,206) and accompanied by a broad margin of error (45,000 – 1.9 mya; Table 4). These estimates place the divergence between the northern and southern populations in the Pleistocene. Second, the migration rates (m1 and m2) between the northern and southern populations are high (Fig. 4). The migration rate m1 is the rate per gene per generation from population 1 (north) to population 2 (south) in the coalescent, or, the rate at which genes come into population 1 (north) from population 2 (south) as time moves forward. The migration rate m2 describes gene migration in the opposite direction. The marginal posterior distributions for m1 and m2 show high points around 2.2 and 2.5, respectively (Fig. 4; Table 4). The migration rate for m2 is slightly higher, although the estimation errors for these parameters and the shapes of the distributions are largely overlapping (Table 4; Fig. 4). Finally, the estimate of effective population sizes for the northern population (q0; 53,292) is slightly lower compared to that of the southern population (q1; 76,633; Table 4). The estimate for the ancestral population (q2; 40,945) is lower and the error around this estimate is high (Table 4).

**Table 3:** Data and models used in IMa2 analyses.

Locus	Sample Size		Length	Model	Mutation Rate(error)
	Northern	Southern			
*Sun10*	80	50	285	HKY	–
*Sun22*	78	58	380	HKY	–
*Sun25*	82	64	129	IS	–
*Sun27*	80	62	152	IS	3.49701×10^−6^(1.5×10^−6^–5×10^−6^)
*Sun28*	32	18	518	IS	–
*Sun31*	80	54	536	HKY	–
*Sun32*	80	56	271	HKY	2.11987×10^−6^ (1×10^−6^ – 4×10^−6^)
*Sun37*	70	56	619	IS	–
*Sun53*	62	56	398	IS	–
*Sun54*	68	46	39	IS	–
*Sun59*	82	62	64	IS	–
*Sun60*	66	58	127	IS	–

Samples with assignment probabilities < 95% are excluded from the analysis.

Mutation rates from [23].

**Table 4:** Results of IMa2 analyses.

	t	q0	q1	q2	m1	m2
Min. Bin	5,554	7,251	7,568	631	0.005	0.006
Max. Bin	2,017,729	1,081,073	1,261,081	1,261,081	9.020	11.640
High Point	158,923	53,292	76,633	40,945	2.219	2.530
Mean	696,206	62,122	83,809	311,348	2.429	2.748
95% Low	45,459	33,747	46,035	11,367	1.196	1.038
95% High	1,942,120	105,667	135,910	1,184,529	4.207	5.290

Values (in demographic units) are averages from the marginal posterior distributions from four runs using different starting seeds.

The generation time in years specified on command line at runtime is 1.0.

The geometric mean of mutation rates per year is 2.722720e-06.

m1 is the rate per gene per generation from population 1 (north) to population 2 (south) in the coalescent, or, the rate at which genes come into population 1 (north) from population 2 (south) as time moves forward.

There are critical limitations to consider when applying the isolation-with-migration method to estimate population parameters in a hybrid zone such as the one studied here. The typical hybrid zone sampling scheme (as used here) ignores the majority of the geographic ranges of lineages and focuses solely on peripheral populations interacting at the hybrid zone. This will tend to violate the major overall assumptions of the isolation and migration model, which include 1) there should be no other populations that are more closely related to the sampled populations than they are to each other, and 2) there should be no gene exchange with other unsampled populations [13], [14]. In this study, the hybrid zone sampling transect ignores the larger patterns of population substructure and phylogeography that describe *Sceloporus tristichus* [4], [7], [8]. Future studies of population divergence in the *undulatus* species group should aim for the inclusion of comprehensive species sampling and range-wide population sampling to better account for gene flow and introgression.

## Materials and Methods

### Multi-locus nuclear data

I collected sequence data from 12 nuclear loci for 93 specimens of *Sceloporus* from throughout the hybrid zone on the Mogollon Rim, Arizona (Appendix 1). Multi-locus data were collected for specimens that were included in a previous analysis of the hybrid zone [4], and therefore have accompanying morphological, chromosomal, and mtDNA data (Appendix 1; see below). The 11 localities sampled span approximately 75 km with an emphasis on a north-south transect following Silver Creek (Fig. 1). The number of samples included from each locality ranges from four (Dry Lake and Woodruff) to 24 (Canoncito) (Fig. 1).

The 12 nuclear loci are anonymous markers isolated from a genomic library constructed from two individuals of *S. cowlesi* from New Mexico [15]. To avoid any possible ascertainment bias that could result from genotyping single nucleotide polymorphisms individually, each locus was resequenced for every individual. PCR protocols are provided by [15]. I sequenced all loci in both directions using an ABI 3730 capillary sequencer. Contiguous DNA sequences were aligned and edited using Sequencher v4.8.

Anonymous nuclear loci are advantageous for phylogeographic studies, because they have the potential to contain a large number of variable sites [16]. However, the non-coding nature of these genomic regions makes them susceptible to insertions and deletions (indels). Although indels provide a rich source of variation in addition to typical nucleotide substitution, individuals that are heterozygous for a particular indel will produce overlapping sequence chromatographs that represent the two length-variant alleles when using standard Sanger sequencing methods. I resolved the length of these heterozygous indels using CodonCode Aligner v2.0.4 (CodonCode Corporation), which can separate overlapping trace files. I truncated the nuclear loci to include only the first heterozygous indel encountered in the forward direction. Under some circumstances, I used CodonCode Aligner to resolve the length of heterozygous indels and recover the downstream sequences to provide longer useable sequence reads.

The gametic phase of genotypes was resolved computationally using Phase v2.1 [17], which incorporates recombination into the model [18]. Only those alleles with reconstruction probability values ≥ 0.95 were used in subsequent analyses. I tested for intra-genic recombination using the difference of sums of squares (DSS) test in TOPALi v2.5 [19]. I calculated F_st_ values and Tajima's *D* neutrality statistic [20] using DNASP v5.1 [21].

### Population structure

Population structure was estimated from the multi-locus nuclear data using Structure v2.2 [9] and Structurama v2.0 [10]. Structure uses the multi-locus genotype data to cluster individuals while minimizing Hardy-Weinberg disequilibrium and gametic phase disequilibrium between loci within groups [9], [22]. The allele frequencies of different populations were assumed to be correlated, which is a reasonable model for populations that are likely to be similar due to migration and/or shared ancestry [22]. An admixture model of individual ancestry was used, which assumes that a fraction of each individual's genome is drawn from *K* population clusters. This is a flexible model that offers a natural way to deal with assigning hybrid individuals into population clusters [9]. The MCMC analyses for each Structure analysis (from *K* = 1 to *K* = 5) was run for 2,000,000 steps after an initial burn-in period of 50,000 steps. For the STRUCTURAMA v. 2.0 analyses [10], the number of populations (K) was treated as a random variable with a Dirichlet process prior (DPP). We ran analyses with the number of populations following a DPP with a prior mean of *E*(*K*) = 2, 4 and 6. All MCMC analyses were run twice with different starting seeds for a total of 1 million generations with sampling at intervals of 1000 steps. The first 100 samples were excluding 100 as burn-in.

### Spatially-explicit population structure

In order to detect population subdivisions that take into account the spatial position of sampled genotypes, I used the Bayesian MCMC program Geneland [11], [12]. Using multi-locus genotype data in conjunction with geo-referenced specimens, Geneland determines the number of population subdivisions in the sample, conducts individual population assignments, and produces a graphical output of the spatial distribution of populations [11]. Assumptions of the model include Hardy-Weingberg equilibrium within loci, linkage equilibrium between loci, and immigrant genes are derived from new immigrants [11]. Two analyses were performed on the multi-locus data. The first analysis identified the most probably number of population clusters, as inferred by the posterior density of population clusters (from 1 to 5) visited during the MCMC analysis. The second analysis was conducted with the number of population clusters set to the number of clusters with the highest posterior density from step one. The MCMC analyses were replicated twice, and each analysis was run for 1,000,000 generations.

### Population divergence genetics

The population divergence genetics parameters of interest were estimated in a Bayesian framework using the isolation-with-migration program IMa2 [13]. All 12 nuclear loci were used to estimate parameters, including the effective population size of each population and the ancestral population (θ1, θ2 and θA), asymmetrical migration rates (m1 and m2), and the time of population splitting (t). The two populations identified in the population structure analyses were used in the IMa2 analyses, and only samples with assignment probabilities ≥ 95% were included.

The infinite sites (IS) model was applicable to eight loci, and the HKY model of nucleotide substitution was applied to the four loci that violated the IS model and contained sites with > 2 character states (Table 3). The autosomal nuclear genes were assigned an inheritance scalar of 1.0. We used the following settings for the prior distributions on population parameters: scalars for theta values  =  10; maximum migration rates  =  20; maximum time of population splitting t = 4. Each analysis incorporated 50 concurrent chains per with geometric heating (g1 = 0.99 and g2 = 0.92). A total of 100,000 steps were retained, with a step length of 20 and the first 100,000 steps discarded as burn-in. The analyses were repeated four times with different random starting seeds to check for consistency across independent analyses. Parameter estimates were rescaled to demographic units at runtime using mutation rates for two of the loci (Table 3).

## Supporting Information

Table S1 **Locality data and voucher specimen information for samples included in the study.**
(DOC)
