# Supplementary material for: Multi-Locus Estimates of Population Structure and Migration in a Fence Lizard Hybrid Zone
Source: PLoS One. 2011 Sep 29;6(9):e25827. doi: 10.1371/journal.pone.0025827 (PMC3183087; doi:10.1371/journal.pone.0025827)
Supplement: Table S1 — Locality data and voucher specimen information for samples included in the study. (DOC) [file pone.0025827.s001.doc]

**Table S1**. Locality data and voucher specimen information for samples included in the study. The assignment of each individual into groupings based on the mtDNA gene tree (four groups: *S. tristichus* North, South, and West; *S. cowlesi*), chromosome seven polymorphism (three karyotypes: SM, submetacentric; ST, subtelocentric; T, telocentric), and morphology (two phenotypes: grassland and juniper) are indicated.

| **Locality** | **Latitude** | **Longitude** | **MtDNA Clade** | **Karyotype** | **Phenotype** |
| --- | --- | --- | --- | --- | --- |
| **Holbrook (9)** | |  |  |  |  |
| AMNH 153945 | 34.90480556 | -110.1944444 | *S. tristichus (North)* | Both SM | grassland |
| AMNH 153946 | 34.90480556 | -110.1944444 | *S. tristichus (North)* | Both ST | grassland |
| AMNH 153948 | 34.90480556 | -110.1944444 | *S. tristichus (North)* | Both SM | grassland |
| AMNH 153950 | 34.92005556 | -110.1564167 | *S. tristichus (North)* | SM + ST | grassland |
| AMNH 153952 | 34.92005556 | -110.1564167 | *S. tristichus (North)* | Both SM | grassland |
| AMNH 153953 | 34.89966667 | -110.1513333 | *S. tristichus (North)* | Both SM | grassland |
| AMNH 153954 | 34.89966667 | -110.1513333 | *S. tristichus (North)* | Both SM | grassland |
| AMNH 153951 | 34.92005556 | -110.1564167 | *S. tristichus (North)* | Both SM | − |
| AMNH 153949 | 34.92005556 | -110.1564167 | *S. tristichus (South)* | Both SM | grassland |
| **Fivemile Wash (8)** | |  |  |  |  |
| AMNH 154017 | 34.8381 | -110.1447 | *S. cowlesi* | Both SM | grassland |
| AMNH 154019 | 34.8381 | -110.1447 | *S. cowlesi* | SM + ST | grassland |
| AMNH 154021 | 34.8381 | -110.1447 | *S. cowlesi* | Both SM | grassland |
| AMNH 154022 | 34.8381 | -110.1447 | *S. cowlesi* | SM + ST | grassland |
| AMNH 154023 | 34.8381 | -110.1447 | *S. cowlesi* | Both SM | grassland |
| AMNH 154015 | 34.8381 | -110.1447 | *S. tristichus (North)* | Both SM | grassland |
| AMNH 154018 | 34.8381 | -110.1447 | *S. tristichus (North)* | SM + ST | grassland |
| AMNH 154020 | 34.8381 | -110.1447 | *S. tristichus (North)* | Both SM | grassland |
| **Washboard Wash (9)** | |  |  |  |  |
| AMNH 153969 | 34.79291667 | -110.0987333 | *S. cowlesi* | Both SM | grassland |
| AMNH 153970 | 34.79291667 | -110.0987333 | *S. cowlesi* | Both SM | grassland |
| AMNH 153968 | 34.79291667 | -110.0987333 | *S. tristichus (North)* | SM + ST | grassland |
| AMNH 153972 | 34.79291667 | -110.0987333 | *S. tristichus (North)* | Both SM | grassland |
| AMNH 153973 | 34.79291667 | -110.0987333 | *S. tristichus (North)* | SM + ST | grassland |
| AMNH 153974 | 34.79291667 | -110.0987333 | *S. tristichus (North)* | Both SM | grassland |
| AMNH 153975 | 34.79291667 | -110.0987333 | *S. tristichus (North)* | Both SM | grassland |
| AMNH 153976 | 34.79291667 | -110.0987333 | *S. tristichus (North)* | SM + ST | grassland |
| AMNH 153977 | 34.79291667 | -110.0987333 | *S. tristichus (North)* | Both ST | grassland |
| **Woodruff (4)** | |  |  |  |  |
| AMNH 153928 | 34.73805556 | -110.0375556 | *S. tristichus (North)* | Both SM | grassland |
| AMNH 153929 | 34.71233333 | -110.0332222 | *S. tristichus (North)* | Both SM | grassland |
| AMNH 153930 | 34.71233333 | -110.0332222 | *S. tristichus (North)* | Both ST | grassland |
| AMNH 153996 | 34.7252 | -110.0281 | *S. tristichus (North)* | − | grassland |
| **Canoncito (24)** | |  |  |  |  |
| AMNH 153892 | 34.66919444 | -110.0416389 | *S. cowlesi* | SM + ST | grassland |
| AMNH 153898 | 34.66780556 | -110.0379444 | *S. cowlesi* | Both SM | grassland |
| AMNH 153917 | 34.66783333 | -110.039 | *S. cowlesi* | Both SM | grassland |
| AMNH 153893 | 34.66880556 | -110.0419722 | *S. cowlesi* | Both SM | juniper |
| AMNH 153902 | 34.66755556 | -110.0363611 | *S. cowlesi* | Both SM | juniper |
| AMNH 153903 | 34.66775 | -110.0377222 | *S. cowlesi* | Both SM | − |
| AMNH 153924 | 34.66744444 | -110.0398333 | *S. cowlesi* | Both SM | − |
| AMNH 153901 | 34.66755556 | -110.0363611 | *S. tristichus (North)* | Both ST | grassland |
| AMNH 153905 | 34.66802778 | -110.0389444 | *S. tristichus (North)* | SM + ST | grassland |
| AMNH 153906 | 34.66802778 | -110.0389444 | *S. tristichus (North)* | SM + ST | grassland |
| AMNH 153909 | 34.66805556 | -110.0398889 | *S. tristichus (North)* | Both SM | grassland |
| AMNH 153912 | 34.66922222 | -110.04175 | *S. tristichus (North)* | Both SM | grassland |
| AMNH 153914 | 34.66755556 | -110.0360833 | *S. tristichus (North)* | Both SM | grassland |
| AMNH 153915 | 34.66811111 | -110.0358056 | *S. tristichus (North)* | Both ST | grassland |
| AMNH 153899 | 34.66772222 | -110.0373611 | *S. tristichus (North)* | SM + ST | juniper |
| AMNH 153907 | 34.66791667 | -110.0385278 | *S. tristichus (North)* | Both SM | − |
| AMNH 153894 | 34.66841667 | -110.0404722 | *S. tristichus (South)* | Both SM | grassland |
| AMNH 153896 | 34.66802778 | -110.0390278 | *S. tristichus (South)* | SM + ST | grassland |
| AMNH 153913 | 34.66919444 | -110.04175 | *S. tristichus (South)* | SM + ST | grassland |
| AMNH 153922 | 34.66769444 | -110.0426944 | *S. tristichus (South)* | SM + ST | grassland |
| AMNH 153923 | 34.66688889 | -110.0438611 | *S. tristichus (South)* | SM + ST | grassland |
| AMNH 153925 | 34.66744444 | -110.0368056 | *S. tristichus (South)* | SM + T | grassland |
| AMNH 153897 | 34.66780556 | -110.0379444 | *S. tristichus (South)* | SM + ST | juniper |
| AMNH 153920 | 34.6685 | -110.0422222 | *S. tristichus (South)* | Both SM | juniper |
| **North Snowflake (8)** | |  |  |  |  |
| AMNH 153982 | 34.6025 | -110.0624 | *S. cowlesi* | SM + T | − |
| AMNH 153984 | 34.6025 | -110.0624 | *S. tristichus (West)* | Both T | grassland |
| AMNH 153983 | 34.6025 | -110.0624 | *S. tristichus (West)* | SM + T | juniper |
| AMNH 153988 | 34.6025 | -110.0624 | *S. tristichus (North)* | Both T | grassland |
| AMNH 153981 | 34.6025 | -110.0624 | *S. tristichus (North)* | SM + T | juniper |
| AMNH 153985 | 34.6025 | -110.0624 | *S. tristichus (North)* | Both T | juniper |
| AMNH 153986 | 34.6025 | -110.0624 | *S. tristichus (North)* | Both T | juniper |
| AMNH 153987 | 34.6025 | -110.0624 | *S. tristichus (North)* | Both T | juniper |
| **Dry Lake (4)** | |  |  |  |  |
| AMNH 153938 | 34.68172222 | -110.3496944 | *S. tristichus (South)* | Both T | juniper |
| AMNH 153941 | 34.68172222 | -110.3496944 | *S. tristichus (South)* | Both ST | juniper |
| AMNH 153942 | 34.68172222 | -110.3496944 | *S. tristichus (South)* | − | juniper |
| AMNH 153940 | 34.68172222 | -110.3496944 | *S. tristichus (South)* | Both T | − |
| **Jct. 180 (6)** | |  |  |  |  |
| AMNH 154052 | 34.61927778 | -109.6621944 | *S. cowlesi* | ST + T | grassland |
| AMNH 154059 | 34.61927778 | -109.6621944 | *S. cowlesi* | Both T | grassland |
| AMNH 154061 | 34.61927778 | -109.6621944 | *S. cowlesi* | ST + T | grassland |
| AMNH 154057 | 34.61927778 | -109.6621944 | *S. cowlesi* | ST + T | juniper |
| AMNH 154058 | 34.61927778 | -109.6621944 | *S. cowlesi* | Both ST | juniper |
| AMNH 154060 | 34.61927778 | -109.6621944 | *S. cowlesi* | Both ST | juniper |
| **Concho (5)** | |  |  |  |  |
| AMNH 154047 | 34.53218333 | -109.7696333 | *S. cowlesi* | Both T | grassland |
| AMNH 154048 | 34.53218333 | -109.7696333 | *S. cowlesi* | Both T | grassland |
| AMNH 154064 | 34.53218333 | -109.7696333 | *S. cowlesi* | Both T | grassland |
| AMNH 154049 | 34.53218333 | -109.7696333 | *S. tristichus (South)* | Both T | juniper |
| AMNH 154050 | 34.53218333 | -109.7696333 | *S. tristichus (South)* | Both T | − |
| **Snowflake (7)** | |  |  |  |  |
| AMNH 154026 | 34.52788889 | -110.0807222 | *S. tristichus (West)* | Both T | juniper |
| AMNH 154028 | 34.52788889 | -110.0807222 | *S. tristichus (West)* | Both T | juniper |
| AMNH 154032 | 34.52788889 | -110.0807222 | *S. tristichus (West)* | Both T | juniper |
| AMNH 154030 | 34.52788889 | -110.0807222 | *S. tristichus (North)* | ST + T | grassland |
| AMNH 154033 | 34.52788889 | -110.0807222 | *S. tristichus (South)* | Both T | grassland |
| AMNH 154027 | 34.52788889 | -110.0807222 | *S. tristichus (South)* | Both ST | juniper |
| AMNH 154034 | 34.52788889 | -110.0807222 | *S. tristichus (South)* | ST + T | juniper |
| **Show Low (9)** | |  |  |  |  |
| AMNH 153956 | 34.33527778 | -110.1033889 | *S. tristichus (South)* | Both T | juniper |
| AMNH 153957 | 34.33527778 | -110.1033889 | *S. tristichus (South)* | Both T | juniper |
| AMNH 153959 | 34.33527778 | -110.1033889 | *S. tristichus (South)* | Both T | juniper |
| AMNH 153962 | 34.33527778 | -110.1033889 | *S. tristichus (South)* | Both T | juniper |
| AMNH 153963 | 34.33527778 | -110.1033889 | *S. tristichus (South)* | Both T | juniper |
| AMNH 153965 | 34.33527778 | -110.1033889 | *S. tristichus (South)* | Both T | juniper |
| AMNH 153966 | 34.33527778 | -110.1033889 | *S. tristichus (South)* | Both T | juniper |
| AMNH 153967 | 34.33527778 | -110.1033889 | *S. tristichus (South)* | Both T | juniper |
| AMNH 153958 | 34.33527778 | -110.1033889 | *S. tristichus (South)* | Both T | − |
